# Supplementary figures and images for: Introgression and mating patterns between white-handed gibbons (Hylobates lar) and pileated gibbons (Hylobates pileatus) in a natural hybrid zone
Source: PLoS One. 2022 Mar 31;17(3):e0264519. doi: 10.1371/journal.pone.0264519 (PMC8970389; doi:10.1371/journal.pone.0264519)

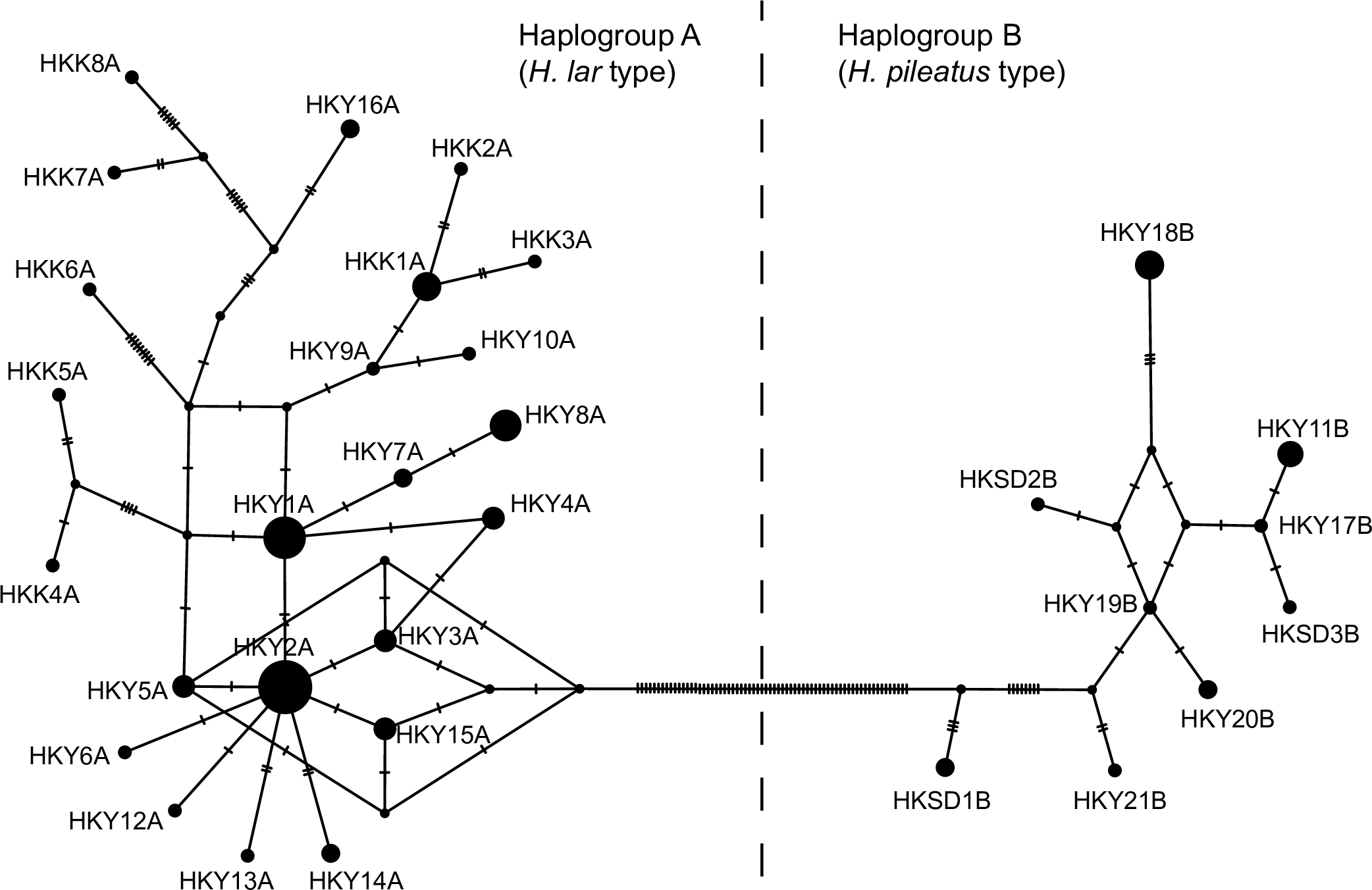

Supplement: S1 Fig — Short bars on branches indicate the number of substitutions between nodes. Node size reflects the number of each haplotype observed among 88 gibbons. HKY: 21 haplotypes observed at Khao Yai; HKK: 8 haplotypes observed at Kaneg Krachan; HKSD: 3 haplotypes observed at Khao Soi Dao. (TIF) [file pone.0264519.s001.tif]

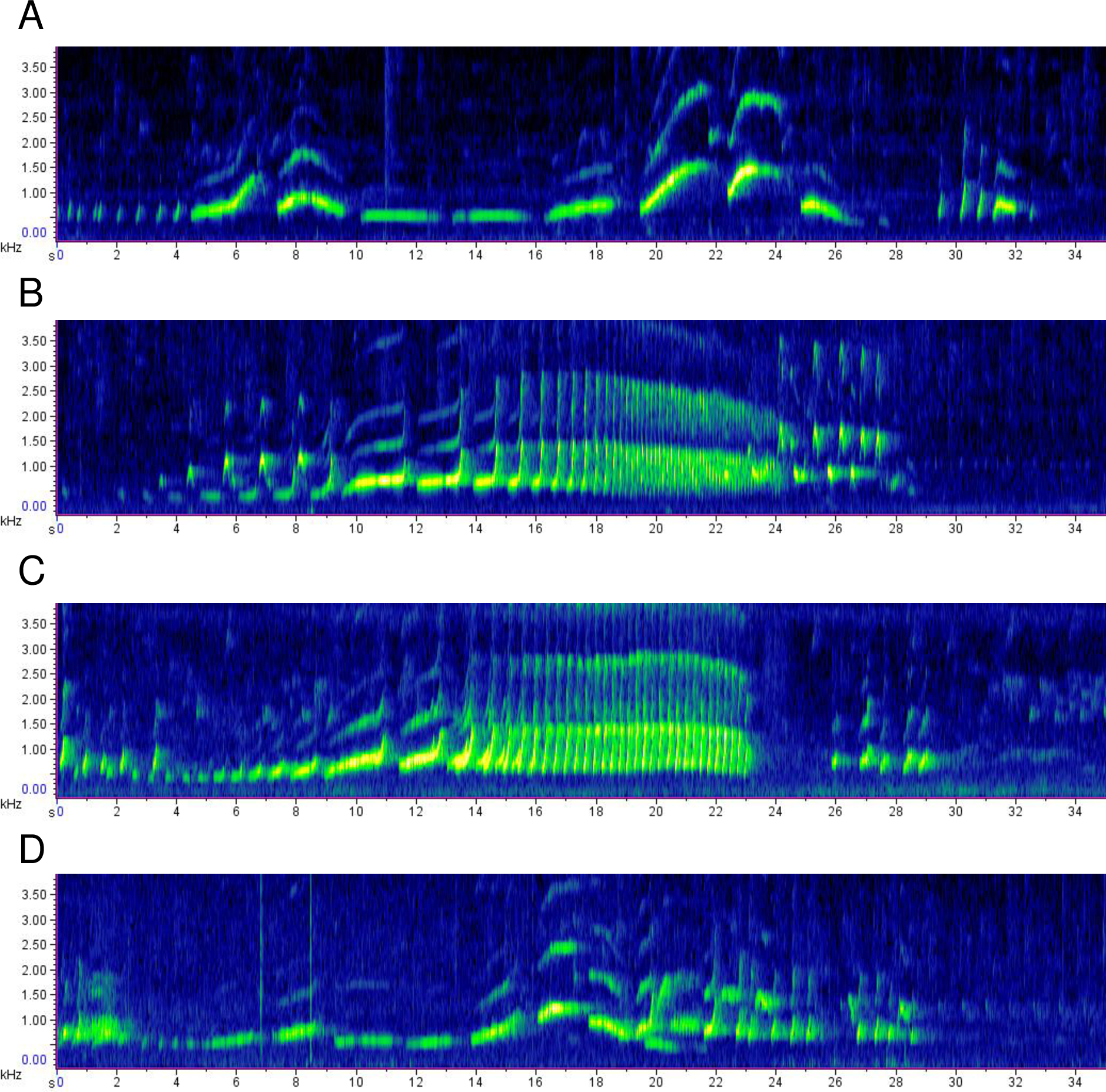

Supplement: S2 Fig — (A) A white-handed gibbon pair at Kaeng Krachan. (B) A pileated gibbon pair at Khao Soi Dao. (C) A hybrid female and a white-handed male pair at Khao Yai. (D) A white-handed female and a hybrid male pair at Khao Yai. (TIF) [file pone.0264519.s002.tif]

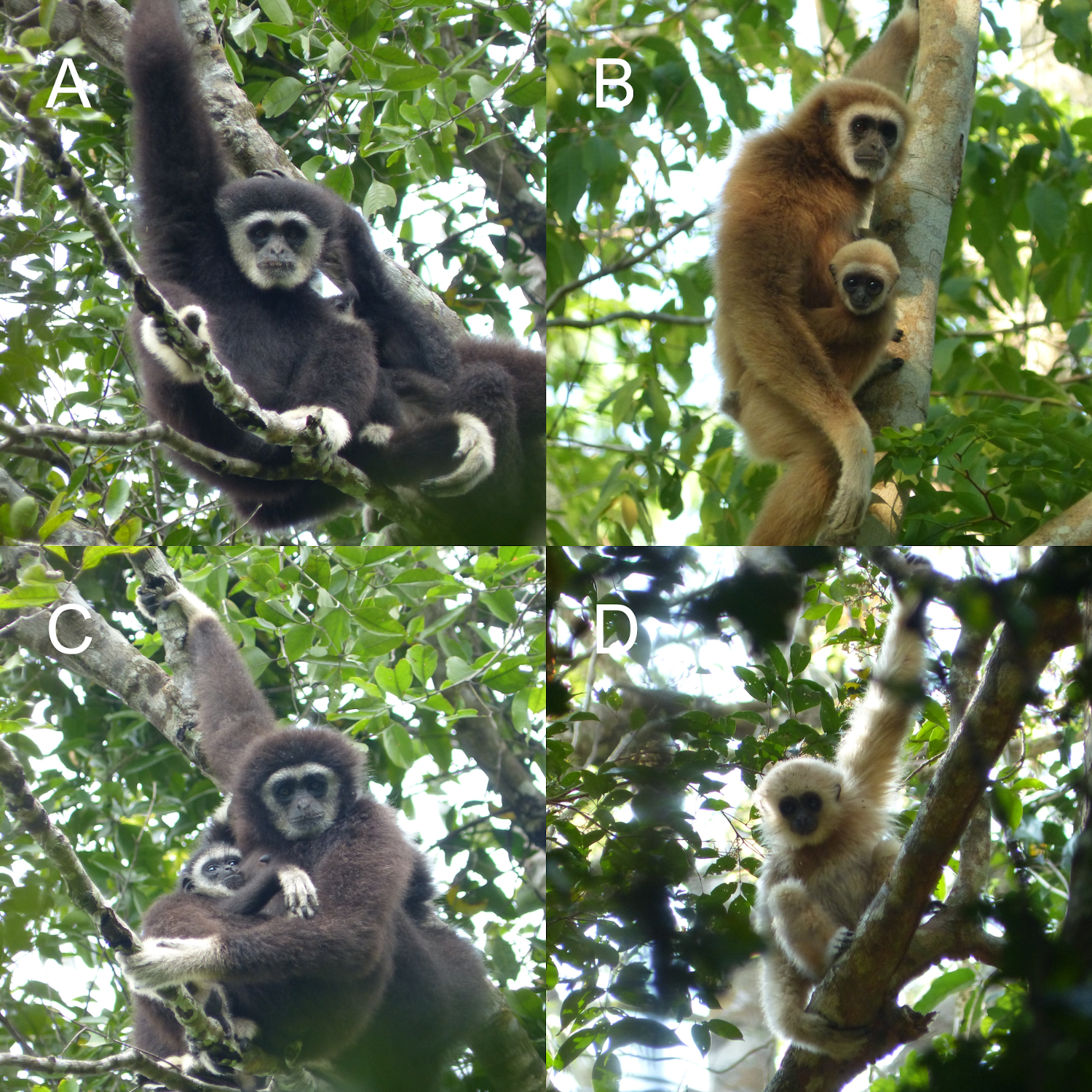

Supplement: S3 Fig — (A) Adult male (black morphotype). (B) Adult female (buff morphotype) and infant (buff morphotype). (C) Adult female (black morphotype) and infant (black morphotype). (D) Juvenile (buff morphotype). (TIF) [file pone.0264519.s003.tif]

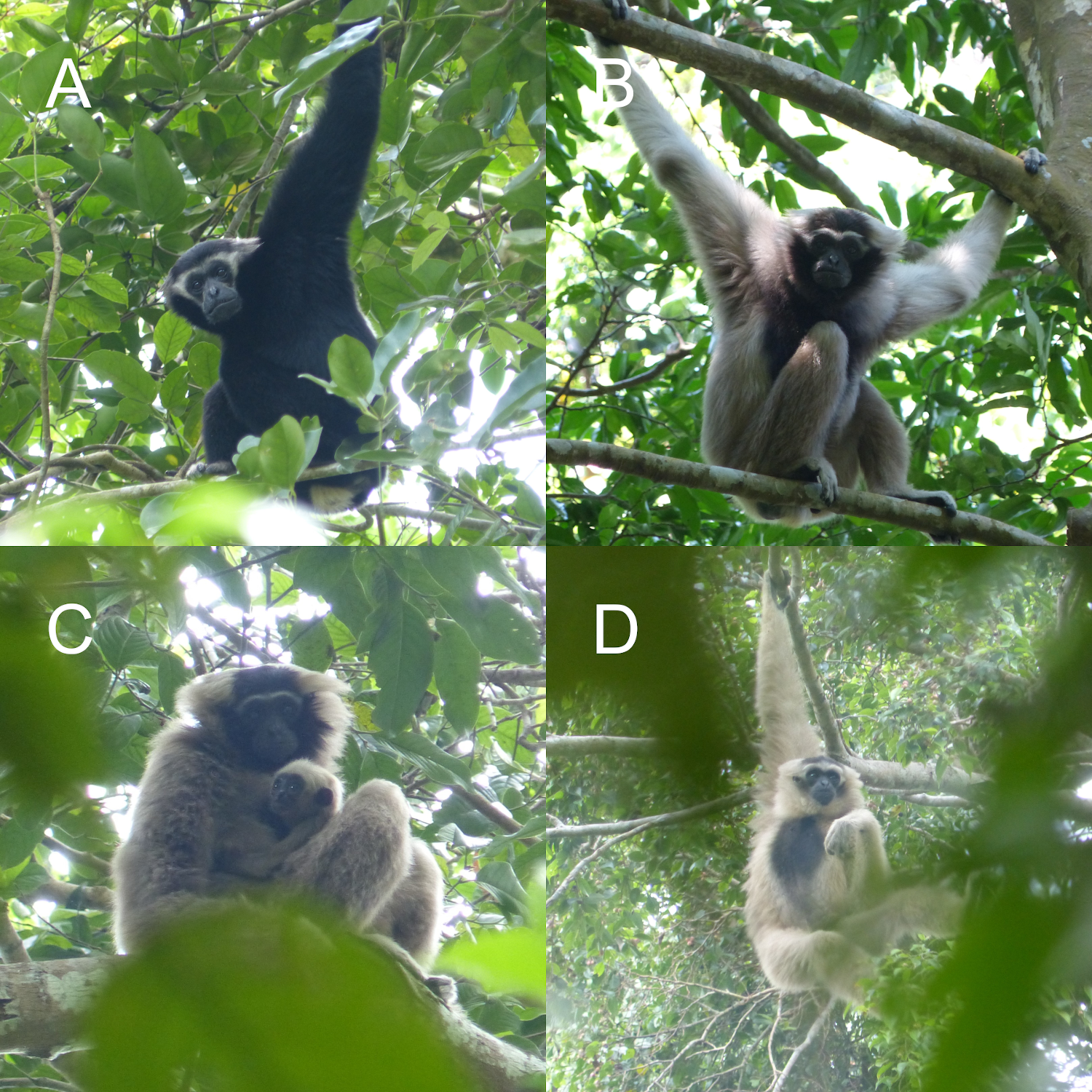

Supplement: S4 Fig — (A) Adult male. (B) Adult female. (C) Adult female and infant. (D) Adolescent. (TIF) [file pone.0264519.s004.tif]

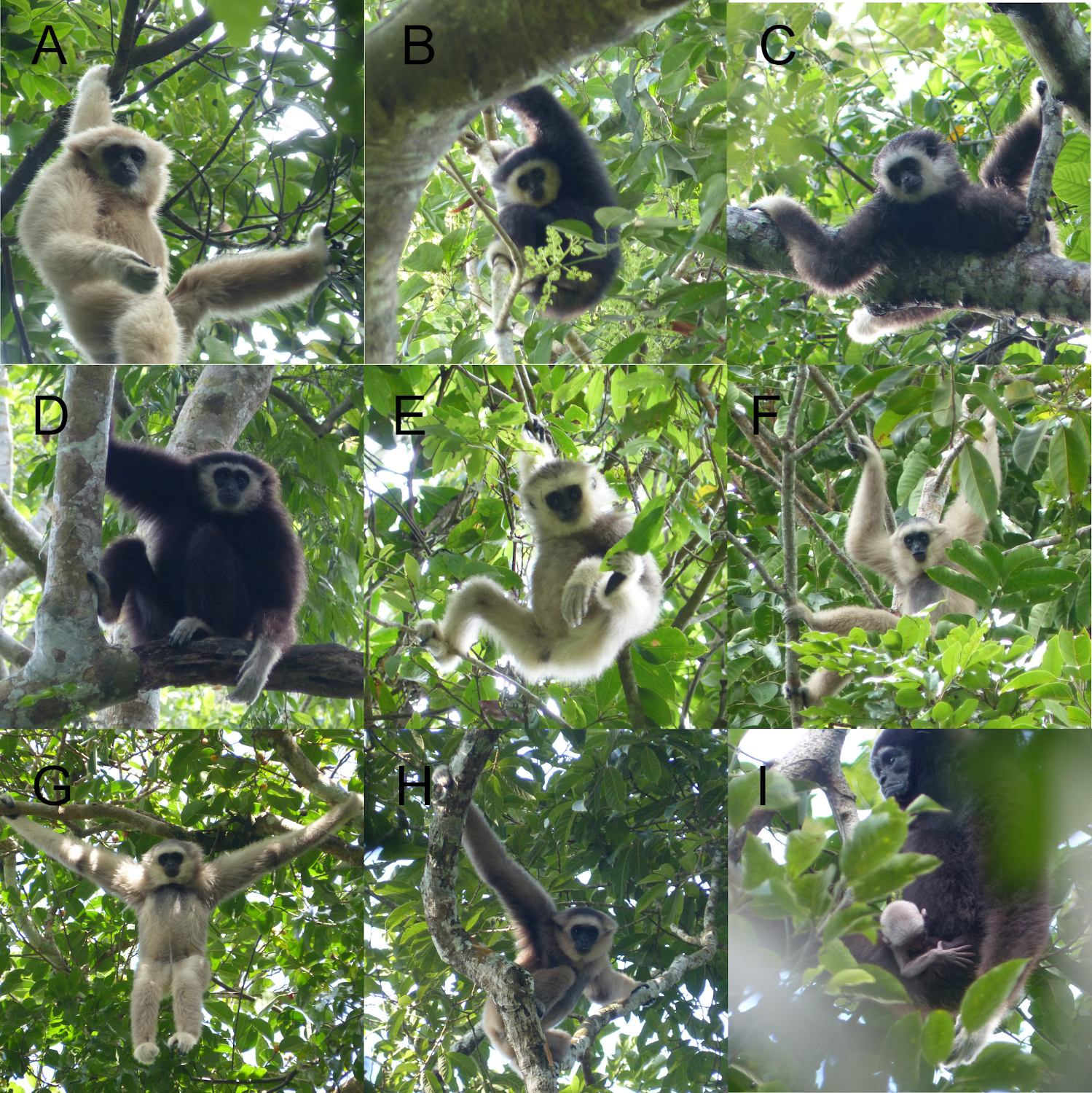

Supplement: S5 Fig — (A) Subadult male of AA1. (B) Adolescent female of AA1. (C) Juvenile female of AA1 (A–C: putative mother = intermediate hybrid). (D) Subadult male of AA2. (E) Juvenile of AA2 (D–E: putative father = intermediate hybrid). (F) Juvenile of AB1 (putative mother = intermediate hybrid). (G) Adolescent female of AC3. (H) Juvenile of AC3. (I) Infant of AC3 (G–I: putative mother = hybrid with a low level of mixed ancestry). (TIF) [file pone.0264519.s005.tif]
