# Supplementary material for: Introgression and mating patterns between white-handed gibbons (Hylobates lar) and pileated gibbons (Hylobates pileatus) in a natural hybrid zone
Source: PLoS One. 2022 Mar 31;17(3):e0264519. doi: 10.1371/journal.pone.0264519 (PMC8970389; doi:10.1371/journal.pone.0264519)
Supplement: Attachment — Submitted filename: Response_to_Reviewers_20211115.docx [file pone.0264519.s008.docx]

Dear Dr. Gojobori,

Thank you very much for evaluating the manuscript entitled “Introgression and mating patterns between white-handed gibbons (*Hylobates lar*) and pileated gibbons (*Hylobates pileatus*) in a natural hybrid zone”. We revised the manuscript following your comments and the two reviewers. The major point is that we toned down the conclusion on the level of admixture of each individual due to the limited number of SNV markers. Please find the details of revisions in the replies below.

In addition, submitting the agreements from copyright holders of the map information for Fig1 and Fig2 were requested. However, we recreated the maps by using only the data provided under the CC BY-SA license and describe the information in the figure legends appropriately. Therefore, we believe the agreements are not needed for these cases.

Comments from the editor

Thank you for submitting your manuscript to PLOS ONE. After careful consideration, we feel that it has merit but does not fully meet PLOS ONE’s publication criteria as it currently stands. Therefore, we invite you to submit a revised version of the manuscript that addresses the points raised during the review process.

Both of the reviewers agree on that the limited number of genetic markers in this study cannot provide enough resolution to clearly distinguish F1s and backcrossed individuals or to evaluate the degree of gene flow. I have to say that the conclusions are not fully justified by the data in the current manuscript. However, I agree with the reviewer #1 on that this manuscript is publishable if the authors can revise the manuscript and tone down the conclusion. Therefore, my decision is “Major Revision”.

There was a disagreement between the reviewers regarding the limited number of available SNPs or genetic markers. Considering that obtaining larger sample size than the current data is difficult, I am more inclined to agree with the reviewer #1. But as reviewer #2 suggested, please explain the difficulty of obtaining samples and justify the reason why the only the limited sample size and the limited number of genetic markers were used.

Answer: Thank you very much for your kind understanding on the difficulties in obtaining large number of both samples and appropriate markers. We revised the manuscript and added the explanation of difficulties in the sampling from unhabituated gibbons (line 187-196) and finding markers (line 211-234). We also agree with you and reviewer #1 at the point of toning down the conclusion because of the difficulties in distinguishing first filial (F1) and backcrosses. Therefore, we avoided concluding the individuals as F1 by replacing the sentences.

There was some unclear points on how the genetic markers were selected and which individuals are included in this study. Please clarify these points on the revised manuscript because these information is essential to make the manuscript technically sound.

Answer: We revised and added detailed information about the finding of markers (line 211-234).

Also please address the other points raised by the reviewers than I mentioned below.

Answer: We revised accordingly. Please find replies below for the details.

Review Comments to the Author

Reviewer #1: Matsudaira and colleges present genetic and phenotypic evidence for hybridisation of white-handed and pileated gibbons in a hybrid zone.

Being unfamiliar with gibbon phenotypic diversity I restrict my comments to the genetic analysis which is within my area of expertise.

The authors present a genetic analysis, using 8 autosomal, MT and Y markers. This appears a novel investigation of a recent and/or ongoing hybridisation of these two species, and a counterpart to the investigation of older introgression described in Matsudaira and Ishida Heredity volume 127, pages312–322 (2021).

As such, I would be pleased if ultimately this paper was published in PLoS One. There is however a question as to how strong the interpretation of the genetic data is.

I certainly would agree with the authors that even a small number of suitably chosen loci can readily differentiate between two diverged lineages, and that these would also provide some information as to introgression/hybrid formation and/or a cline of ancestry. I disagree that there is enough resolution to readily differentiate between F1/F2 or various generations of repeat backcrosses. Given the small number of autosomal markers (less than the number of chromosomes), and the associated uncertainty in estimated ancestry components- even with only K = 2 - many different population processes could result in very similar arrays of ancestry that the authors uncover within the hybrid zone. Thus, I think it is fair to conclude that the authors uncover genetic evidence for recent hybridisation (Recent being relativeto the related findings in Matsudaira and Ishida 2021). I do not think that one can say, as the authors do in the Abstract "Four phenotypically white-handed gibbons showed introgressed pileated type mtDNA, but the autosomal SNV admixture was not detected" and therefore ... "some genomic portions have introgressed into the gene pool of the counterpart species". In my opinion, this is too strong an interpretation of their results. I would be much happier if statements of this kind came with a suitable disclaimer. For example: "These gibbons were not only F1 hybrids, but also ones with plural backcross generations" (Lines 405:406). Much better could be, "Our data suggests that these gibbons could be a mixture of F1 hybrids, and perhaps ones with plural backcross generations, but the limited resolution enabled by small number of marker loci cannot rule out other scenarios acting on shorter timescales, such as a mixture ofF1/F2 individuals". Of course how the authors want to address this point is up to them. Also, please note this is but one example, and my comment should be interpreted as having the authors rethink all statements on genetic ancestry that are definitive in tone such as Lines 428-429.

Answer: Thank you very much for your kind comments on improving the manuscript. We agree with your comments that the small number of SNV markers used in the study has limitations in determining the generation of hybrids. We revised the manuscript and avoid the use of the term, F1, for the individuals who showed intermediate admixture levels. We also toned down the conclusion following the comments.

Minor points:

Data availability. I searched all three databases and could not find the listed accessions. Can the authors please confirm the availability of data.

Answer: The sequences were registered and thus the accession numbers were assigned by DDBJ. Opening the sequences for the public is pending. We will open the data as soon as the manuscript is accepted.

Selection of autosomal SNVs: please expand on the relevant methods. How many DNA sequences from GenBank were initially selected?; how many independent fixed differences did you find? How many additional loci came from Perelman et al?; In essence – the reader should have some clarity about how you came to only 8 SNVs.

Answer: We followed the comments and added detailed explanations for the screening process of SNVs (line 211-234).

Lines 70-71: citation for disadvantages of hybridisation.

Answer: We added the citation and an example of disadvantage (line 67-70).

Reviewer #2: In this manuscript, “Introgression and mating patterns between white-handed gibbons (Hylobates lar) and pileated gibbons (Hylobates pileatus) in a natural hybrid zone”, the authors seek to uncover the degree of introgression and mating patterns between two endangered gibbon species, white-handed and pileated gibbons. They genotyped 88 individuals including 72 individuals from the hybrid zone, 12 purebred white-handed and 4 purebred pileated gibbons, with 8 autosomal SNPs. They also determined 632 bp-sequence on mtDNA and one 2bp-indel on Y chromosome for most of the individuals.

However, as I explained below, I don’t think it’s possible to measure the extent of introgression genetically with this data because of the small number of SNPs and the small number of the purebred samples. Rather, the importance of this study is in proposing potential markers to distinguish the two species and in accumulating genotype information of these endangered species. I recommend to reorganize this manuscript in that way.

Answer: Thank you very much for your kind comments on the manuscript. We agree that our previous conclusion was overstated against the small number of SNV markers used in the study and thus some ambiguity in the resolution of evaluating the admixture level remained. However, as we can see in the results, STRUCTURE analysis successfully distinguished pureblood white-handed gibbons and pileated gibbons without prior information of the species status in these samples (i.e. STRUCTURE did not know which samples were of white-handed gibbons and which samples were of pileated gibbons). Therefore, the finding of the presence of hybrid individuals with various levels of admixture level is robust. Following the comments from the editor, Dr. Gojobori, and reviewer #1, we toned down the conclusion. We believe our revisions made the manuscript scientifically feasible.

1. The major concern is the small number of SNPs analyzed in this manuscript (8 SNPs) and the small number of the purebred individuals (12 white-handed and 4 pileated gibbons). It is hard to say that these SNPs are fixed differences between these species. The authors chose the 8 autosomal SNPs from the previous works of other groups; 5 SNPs from Chan et al. (2013) and 3 SNPs from Perelman et al. (2011). But the numbers of the animals used in the papers are also very small. In Chan et al., 11 white-handed and 4 pileated gibbons were used, and in Perelman et al., only one white-handed gibbon is used. It means, the genotypes of the 8 autosomal SNPs are available only for 13 or 23 white-handed gibbons and 4 or 8 pileated gibbons in total. It is likely that the 8 SNPs are not fixed differences between the species but are segregating at high frequency within each species. In that case, it’s not possible to distinguish the two species with these SNPs.

Answer: Technically saying, genetic markers used in STRUCTURE analysis do not have to be fixed in K species/populations. STRUCTURE assumes the presence of hidden K clusters among the samples, where each cluster has a particular frequency of each SNV. In this process, STRUCTURE does not know which sample comes from which cluster a priori. Therefore, if the number of SNV markers and the number of pureblood white-handed gibbons and pureblood pileated gibbons were too small to distinguish the two species, the membership coefficient shown by STRUCTURE should have not represented the ancestry of the two species. Fortunately, as we can see in the results, 12 gibbons from Kaeng Krachan and 4 gibbons from Khao Soi Dao showed a quite small amount of mixed ancestry which can be considered the noise usually observed in STRUCTURE analysis, and thus (it is safe to say, that) the number of SNV markers and number of pureblood gibbons used in the study was enough for detecting hybrid gibbons with admixed ancestry.

On the other hand, we agree that the number of the SNV markers was not enough to clearly distinguish first filial, second filial and several backcross generations as suggested by the editor and reviewer #1, we revised this point and toned down the conclusion.

2. In lines 269-270 and in Fig 3., the authors mentioned “the threshold of backcross and pure white-handed gibbons”. How was it determined? Also, how the criteria between F1 and backcross was determined (Fig3)? I guess it came from the identification of species base on vocalization and morphology. If so, it means that in this manuscript, the species identification is not based on genetic data but based simply on the vocalization and morphology. The aim of this manuscript is written as analyzing the samples genetically to evaluate the extent of introgression, but the actual analyses are done the other way round. I think the authors’ true interest is in finding a set of potential markers to distinguish species for future studies and to confirm hybrids which are identified from vocalization and morphology. I do understand the importance of finding the markers for endangered species, for which it’s difficult (/not possible) to get good amount of DNA samples. But the manuscript needs to be modified to make the aim clear.

Answer: The threshold of backcross and pureblood white-handed gibbons was determined based on the observed admixed ancestry values in 12 pureblood white-handed gibbons from Kaeng Krachan and 4 pureblood pileated gibbons from Khao Soi Dao. Because of the characteristic of STRUCTURE analysis, a small amount of mixed ancestry could be detected in pureblood samples. Therefore, the maximum admixed ancestry value 0.017 observed in one Kaeng Krachan white-handed gibbon was defined as the threshold. We added an explanation on this point (line 279-287). The threshold between the F1 and backcross was determined arbitrary and thus was inappropriate. Therefore, we revised and removed the distinction between F1 and backcross, and considered all the individuals as mere hybrids.

3. The sample information is not clear enough. The authors collected 129 fecal samples in Khao Yai, 71 fecal samples in Kaeng Krachanand 9 fecal samples on Khao Soi Dao (lines179-181). They also genotyped DNA samples previously collected in Khao Yai (lines 193-194). Then suddenly declared “Overall, 93 DNA samples of 88 individuals were genotyped” without any explanations. Please provide the full sample list with sample ID, sampling location, sex, age category, species identified by vocalization and pelage coloration, and information from genetic data. If the data includes the samples

which were used in previous studies, please explain it with the same sample IDs as in the previous works.

Answer: We restructured the section and made the number of individuals used in the analysis clear (line 187-196). We also added the information of individuals, fecal samples, genotypes etc. as Supplementary Table 2 following the comments from reviewer #2. We hope the additional information will help to understand the details of the subjects.

4. I think the authors should explain about the endangered status of the two species more, if that is the reason why the amount of genetic data is very limited.

Answer: The limitation of sample number is derived from the difficulty in collecting fecal samples from unhabituated gibbons (it took many days to collect fecal samples from each individual). Also, the limitation of the genetic markers was mainly due to the financial issue for designing primers and probes rather than the available sequences from the database.

5. The authors wrote “The electropherograms of HVR1 were visualized by MEGA6 " (line 247). Is it a mistake? I couldn’t find electropherograms in this manuscript.

Answer: Electropherogram is raw data of electrophoresis by a Sanger sequencing and thus usually not informative to be shown in the manuscript without particular reason (e.g. to emphasize the appearance of heterozygous sites in the raw data). It is possible to show an example of one sequencing reaction, but we don’t think it is essential for this manuscript. To avoid misunderstanding what the electropherogram is we revised the sentence and add an explanation (line 265-266).
