# Supplementary material for: Introgression and mating patterns between white-handed gibbons (Hylobates lar) and pileated gibbons (Hylobates pileatus) in a natural hybrid zone
Source: PLoS One. 2022 Mar 31;17(3):e0264519. doi: 10.1371/journal.pone.0264519 (PMC8970389; doi:10.1371/journal.pone.0264519)
Supplement: Attachment — Submitted filename: Response_to_reviewers_20220210.docx [file pone.0264519.s009.docx]

Dear Dr. Gojobori,

Thank you very much for evaluating the revised manuscript entitled “Introgression and mating patterns between white-handed gibbons (*Hylobates lar*) and pileated gibbons (*Hylobates pileatus*) in a natural hybrid zone.” We revised our manuscript following comments of reviewers #3 and #4. A major revision point has been that we now adopt the term “possible hybrids” to describe some more ambiguous results in our analyses. We now also mention the limitation of the use of the small number of SNV loci in our study. We hope this revision now resolves reviewers’ concern of potential overstatement of results and thus we toned down some of our findings. Please find our detailed revisions in the reply to reviewers’ comments below.

Reviewer #3: I agree with both previous reviewers that the limited amount of data presented here should not be over-interpreted. In particular, I agree with the statement 1 of reviewer #2, the previously described SNPs might not represent fixed differences with the small number of individuals, especially since ancient admixture events were likely part of their history.

First, the observation in the STRUCTURE analysis could be explained by genetic clines or simply segregating variation rather than recent hybridization. There is one thing I would recommend in terms of analysis, which is a STRUCTURE analysis with higher k (3, 4, 5) to see if the supposed signature of mixing is not just a component restricted to individuals from this area.

Answer:

We performed STRUCTURE analysis with K = 3, 4, and 5. The analyses result almost equally divided “white-handed-gibbon ancestry” into 2, 3, and 4 components in each individual from the three study sites with no detectable change in “pileated-gibbon ancestry.” We interpret this to indicate that the supposed signature of mixing is not just a component restricted to individuals from Khao Yai National Park. We added the new information and analyses with K = 3, 4, and 5 in Methods and Results sections (Lines 248-253 and lines 273-278, respectively).

Second, the threshold for assigning “hybrid ancestry” seems somewhat low and arbitrary given the small number of loci. Inspecting Fig. 3, there are 5-7 likely hybrids with substantial ancestry from both species (if one assumes these loci are really fixed), while I would be more doubtful about the others. The results on morphology and pelage are lending good support to the hypothesis on observed hybrids, especially in the case of those with clear mixed ancestries. I would like to know if the “five hybrid individuals indistinguishable from typical white-handed gibbons” had a lower assignment of admixed ancestry than the three with atypical patterns (L373).

Answer:

There was no clear relationship between the degree of mixed ancestry and the presence/absence of an atypical pelage color pattern among individuals with low levels of mixed ancestry. We added the explanation in Results (Line 379-383).

Third, the discordance of mtDNA nuclear DNA might be pointed out. There are a few individuals with a haplotype assigned to *H. pileatus* which, in this restricted dataset, have nuclear ancestry only assigned to *H. lar*. This shows the difficulty in determining ancestries with such few markers. Compare that to humans and Neanderthals, where ancient Neanderthal admixture in modern humans has not been determined by obtaining several mtDNA sequences, nor by retrieving a small amount of nuclear DNA (Green et al., Nature 2006), but only by shallow genome sequencing (Green et al., Science 2010). I agree that it is difficult to obtain information from wild-living primates for this kind of studies, but it is a problem that the confidence in observations must be low when using such a small number of individuals for the reference populations and a very small number of genetic markers. In that sense, I would also add “putative” to any statement about admixture, from the abstract on throughout the paper. I.e. the phrasing should be even more cautious when referring to the genetic results.

Answer:

We followed this and the comment of reviewer #4, and revised corresponding sections to read “putative” when our results seemed ambiguous. Because there was no ambiguity in mixed ancestry among intermediate hybrids, we did not use “putative” to describe ancestry of those individuals. For other gibbons with low levels of mixed ancestry, we decided to recategorized them into “hybrids with low levels of mixed ancestry” and “possible hybrids”. We cannot fully exclude that some arbitrariness for making the decision between those two categories may remain, but considering the morphology and vocalization, the hybrid origin of “hybrids with low levels of mixed ancestry” seems apparent to us thus justifying the use of this category. We believe our revisions resolved the overstatement on the admixture of gibbons in this population.

Finally, technological innovations may likely offer opportunities to obtain much more information from fecal samples of wild primates, as has been shown in chimpanzees with sequencing after capture (see Fontsere et al., Molecular Ecology 2021). Considering the very finegrained picture that were obtained in humans and other primate species after sequencing at least substantial parts of the genome, in comparison to single markers or mtDNA sequences, I suggest the authors add a sentence into the discussion about that. This may in the future confirm hypotheses on hybridization gained from the limited data, although of course this also depends on funding.

Answer:

Following the suggestions, we added the limitation of the use of a small number of SNV markers and the prospects of applying new technologies to fecal genomic analysis of the Khao Yai gibbon population (Line: 443-448); we also added suggested references #55 and #56.

Some typos:

L191: fecals > fecal

L212: sentence broken

L317/319: who *were* identified

L349: futher > further

L427: not > to

L435: result*ed*

Answer:

We corrected all typos.

Reviewer #4: This manuscript is about investigating gibbons genetically in the hybrid zones. They make new genetic markers for identifying whether the individual is a “pure-blood” or a “hybrid”. They also discuss on the phenotypic traits for the individuals which were detected as hybrids.

This is a revised manuscript and I was not one of the original reviewers. I will make comments based on the previous reviewers’ comments and the author’s responses.

Overall, the previous comments made by the original reviewers make sense to me and the authors responses are satisfactory. I agree with the previous comments on the limitation of genetic markers. The authors efforts to tone down the conclusion was necessary to make the manuscript more technically sounding.

I have one concern on detecting hybrid using the result of STRUCTURE (e.g. fig 3).

The authors set the criteria for being hybrid as showing contributions of the minor ancestry at a level of more than 0.017. I think it is OK to label the individuals showing “intermediate” level of mixed ancestry as recent hybrids. However, it might be inappropriate to label individuals with marginal value of ancestry (i.e. 0.018 or 0.019) as recent hybrid. This is because the number of markers used is quite limited and even truly “pure-blood” individual may show more than 0.017 of minor ancestry level under this condition. Then I suggest the author to label the individuals with marginal value of ancestry as “possible hybrids” or so.

Answer:

Please, see our comment in response to reviewer #3 above. In sum, we adopted reviewers’ criticism and now introduced “possible hybrids” for individuals with marginal mixed ancestry.
